# Inhibition of thioredoxin reductase 1 by evernic and vulpinic acids: a promising anticancer strategy on A549 cells

**DOI:** 10.1007/s00210-025-04363-w

**Published:** 2025-06-11

**Authors:** Şükran Günaydın, Şeyda Nur Kalın, Emine Karaca Sulukoğlu, Ahmet Altay, Harun Budak

**Affiliations:** 1 Science Faculty, Department of Molecular Biology and Genetics, Atatürk University, 25240 Erzurum, Türkiye; 2 Faculty of Engineering and Natural Sciences, Department of Molecular Biology and Genetics, Kütahya Health Sciences University, 43100 Kütahya, Türkiye; 3 East Anatolia High Technology Application and Research Center, Atatürk University, 25240 Erzurum, Türkiye; 4 Faculty of Science and Arts, Department of Chemistry, Erzincan Binali Yıldırım University, 24002 Erzincan, Türkiye; 5 Science Faculty, Department of Molecular Biology and Genetics, Erzurum Technical University, 25070 Erzurum, Türkiye

**Keywords:** Lung cancer, Evernic acid, Vulpinic acid, Thioredoxin reductase 1, Anticancer activity

## Abstract

TrxR1 (Thioredoxin reductase 1) is overexpressed in several types of cancer, containing lung cancer, which is closely linked to metastasis. Hence, TrxR1 has emerged as a target for cancer therapies. In this study, it was aimed to survey whether lecanoric (LA), evernic acid (EA), and vulpinic acid (VA) exert an anticancer effect on lung cancer (A549) cells by targeting the TrxR1 protein. The findings indicated that VA elicited a more notable cytotoxic activity in A549 cells (36.21 µg/mL) at a lower IC_50_ value than EA (139.09 µg/mL). Whereas, LA exhibited very low cytotoxic effect in A549 cells. The flow cytometry and qPCR analyses revealed that VA was superior to EA in inducing apoptosis and necrosis in A549 cells. Moreover, the wound healing assay revealed that both EA and VA considerably inhibited the migration of A549 cells. Furthermore, EA and VA markedly decreased protein expression and enzyme activity of TrxR1 in A549 cells. All these findings revealed that EA and VA possess anticancer activities by targeting TrxR1 in A549 cells. Hence, EA and VA can be proposed as candidates for use as a potential chemotherapeutic agent for lung cancer.

**Graphical abstract:**

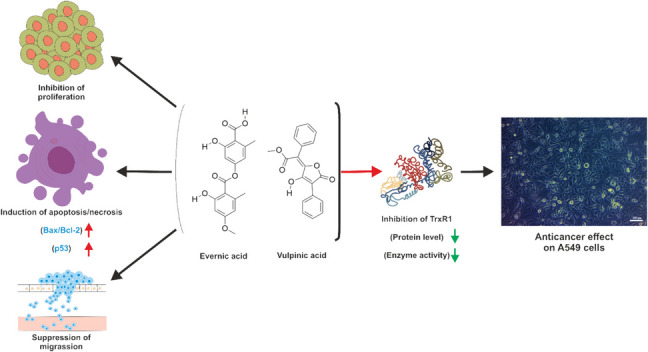

## Introductıon

Lung cancer is one of the most frequently diagnosed cancers in both men and women and is the leading cause of cancer-related death (Chaitanya Thandra et al. 2021). It is estimated that about 2.2 million new cases of lung cancer and roughly 1.8 million deaths related to lung cancer occur all around the world, and these incidence and death rates are predicted to continue to rise rapidly (Chaitanya Thandra et al. 2021). Lung cancers can be histologically separated into two major groups depending on the morphology and appearance of malignant cells. Non-small cell lung cancer (NSCLC) accounts for almost 80–85% of lung cancers (Travis et al. 2015). NSCLC, which is usually diagnosed at an advanced stage, has a poor prognosis with drug resistance outcome and the 5-year survival rate for NSCLC remains below 15% (Testa et al. 2018). Therefore, there is a need to improve treatments for lung cancer patients, to develop more effective natural anticancer agents, and to elucidate the molecular mechanisms underlying these tumors.

A plethora of conventional treatment modalities are available for the management of lung cancer, encompassing surgical interventions, radiotherapy, chemotherapy, and targeted therapy. The selection of these treatment methods is contingent upon the type and stage of the cancer. Notwithstanding advances in diagnosis and treatment, the prognosis for lung cancer patients remains unsatisfactory and responses to current standard therapies can be poor, with the exception of the most localised cancers. A significant point of research is the development of new natural compounds that exhibit high efficacy and minimal or no side effects, given the adverse effects of conventional chemotherapy. In recent years, the focus has been on lichens and their secondary metabolites, which are natural products with anticancer properties (Lemjabbar-Alaoui et al. 2015; Li et al. 2023).

Lichens are organisms with naturally occurring mutualistic relationships between fungi (mycobionts) and algae/cyanobacteria (photobionts). More than a thousand lichen secondary metabolites have been identified in lichens that have been used for medicinal purposes since the past (Molnár and Farkas 2010). Many pharmacological properties of lichen secondary metabolites have been reported up to now, involving anti-inflammatory, antiviral, antimutagenic, antioxidant, and anticancer (Ebrahim et al. 2016; Solárová et al. 2020). Furthermore, natural compounds may exhibit superior antioxidant activity compared to synthetic antioxidants, and lichens also play an important role as a source for new antioxidant substances (White et al. 2014).

A hallmark of cancer is oxidative stress, which results from an imbalance between the antioxidant defense system’s activity and the generation of ROS (reactive oxygen species). ROS are metabolic by-products and extremely reactive molecules that can be beneficial or harmful to cellular components depending on their amount. The low-to-intermediate level of ROS actualizes various functions such as cell growth, proliferation, and cell signaling (Rajavel et al. 2019). However, with the high level of ROS, the redox balance of the cell is disturbed and oxidative stress occurs. After all, it leads to harmful effects on cells in cells like macromolecular damage, cell proliferation, apoptosis, metastatic potential, and carcinogenesis. In that event, the antioxidant system neutralizes ROS to protect cells from free radicals (Sharifi-Rad et al. 2020). Since cancer cells seem to be more sensitive to ROS than normal cells, maintaining ROS homeostasis through natural products can be an effective strategy for treating cancer (Zhang et al. 2017). Thioredoxin system, one of the enzymatic antioxidant systems, catalyzes the NADPH-dependent reduction of the redox protein thioredoxin (Txn) (Fan et al. 2014). TrxR1 (EC 1.8.1.9), a mammalian cytosolic isoform, is overexpressed in many cancers, so it can be considered an attractive approach and a valuable target for the development of anticancer drugs (Fan et al. 2014; Zhang et al. 2017; Rajavel et al. 2019; Jovanović et al. 2020).

A considerable number of phenolic compounds have been demonstrated to possess anticancer properties, and are therefore regarded as potentially valuable therapeutic agents (Belhouala et al. 2024). The phenolic acids lecanoric, evernic, and vulpinic acids are secondary metabolites obtained from lichens. In our previous study, we demonstrated that lecanoric, evernic, diffractaic, lobaric, and vulpinic lichen acids, particularly lecanoric and vulpinic acid, exhibited an inhibitory effect on mitochondrial TrxR purified from rat lung tissue (Ozgencli et al. 2019). Lecanoric acid is a secondary metabolite that has been reported to enhance cytotoxic activity, protect lichen symbionts from light, and exhibit antimicrobial, antioxidant, and fungitoxic properties (Luo et al. 2009). Evernic acid has been shown to have antioxidant, antimicrobial, and antiproliferative properties against different cancer cell lines (Kosanić et al. 2013). Vulpinic acid has been demonstrated to have antiproliferative, antiangiogenic, antimicrobial, and anticancer activities (Koparal 2015; Cansaran-Duman et al. 2021b). Nevertheless, the anticancer effect of lecanoric, evernic, and vulpinic acids on lung cancer cells has not yet been fully elucidated, and the protein target in cells remains unclear. The current research aimed to investigate the cytotoxic, apoptotic, and antimigratory effects of lecanoric, evernic, and vulpinic acids on lung cancer A549 cells and to examine whether this potential effect is mediated by TrxR1 at the level of gene, and protein expressions, as well as enzymatic activity.

## Materıals and methods

### Cell culture condition

The human non-small cell lung cancer cells (A549, RRID:CVCL_0023) for this study were provided by the American Type Culture Collection (ATCC). A549 cells were grown in Ham’s F-12 K (Kaighn’s) medium (Thermo Fisher Scientific), which contained 10% (v/v) heat-inactivated Fetal Bovine Serum (FBS) (HyClone) and 1% penicillin/streptomycin (Thermo Fisher Scientific). The cells were kept at 37 °C in a humidified atmosphere with 5% CO_2_.

### Preparation of test compounds

**Fig. 1:**
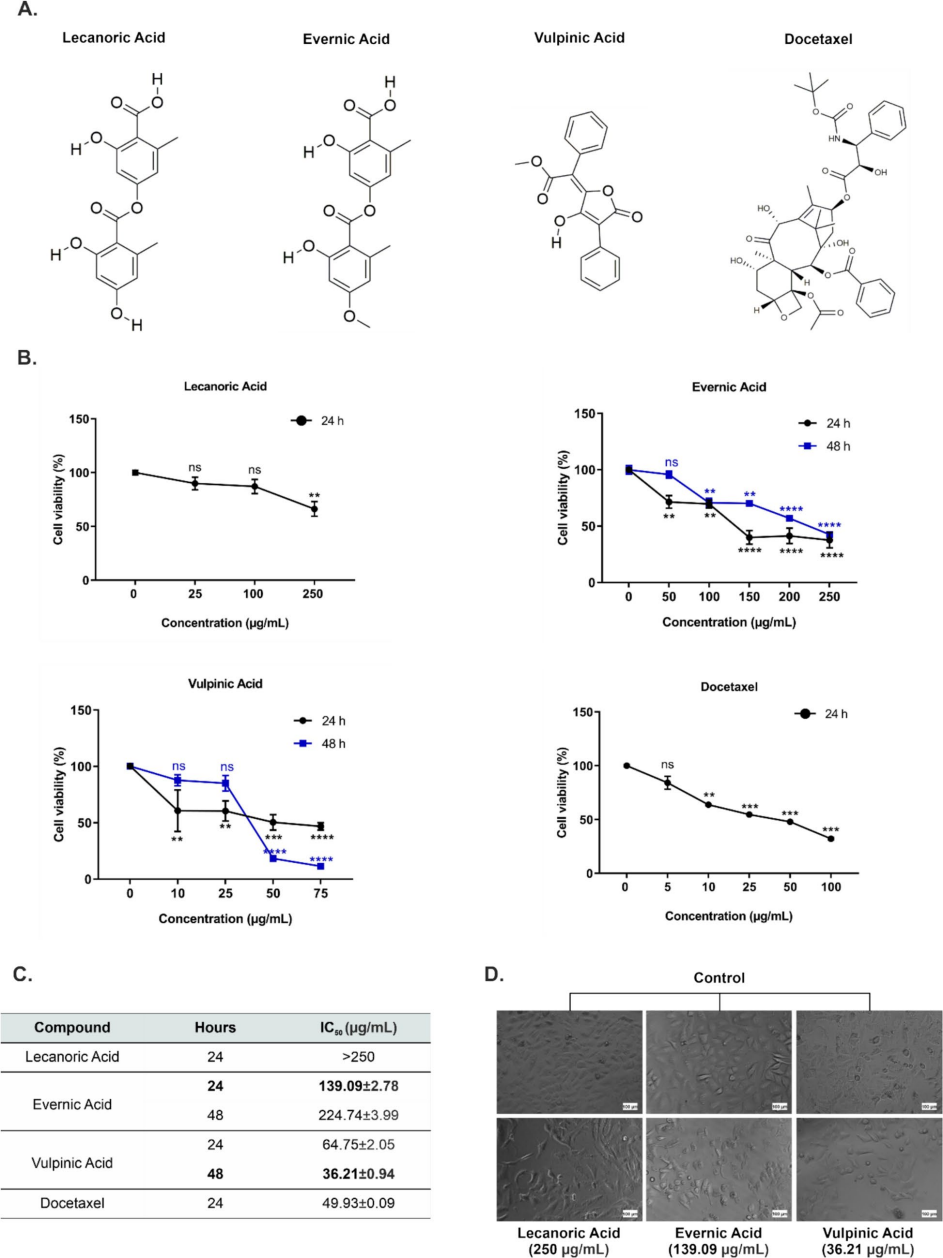
Effect of lecanoric, evernic, and vulpinic acids on the viability of A549 cells. **A** The chemical structures of LA, EA, VA, and the chemotherapeutic drug docetaxel are represented. **B** A549 cells were treated with varying concentrations of lichen acids and docetaxel for 24 and 48 h and the resulting cell viability percentages were determined via the XTT assay. **C** The IC_50_ values of the tested compounds at 24 and 48 h are presented. **D** The effects of lichen acids on A549 cells are illustrated in inverted light microscopy images. Microscope images at 250 µg/mL at 24 h for lecanoric acid, 139.09 µg/mL at 24 h for EA and 36.21 µg/mL at 48 h for VA are presented. The IC_50_ values were presented as the mean ± SD. The graphical data are expressed as the mean ± S.E.M. of three independent experiments. *p* > 0.05 = (ns = not significant), *p < 0.05 significant, **p < 0.01 very significant, ***p < 0.0001, and ****p < 0.0001 extremely significant. Scale bar: 100 μm

Lecanoric acid (C_16_H_14_O_7_), evernic acid (C_17_H_16_O_7_), and vulpinic acid (C_19_H_14_O_5_) were purchased from Cayman Chemical Company (Michigan, USA) and dissolved in dimethyl sulfoxide (DMSO) and kept at −20 °C till used. Docetaxel (C_43_H_53_NO_14_) was obtained from Apollo Scientific (Apollo Scientific Ltd, UK). The chemical structures of vulpinic, evernic, and lecanoric lichen acids as well as docetaxel were given in Fig. 1A. These compounds were diluted with fresh complete Ham’s F-12 K medium to obtain different concentrations of 25–250 µg/mL for lecanoric acid; 50–250 µg/mL for evernic acid; 10–75 µg/mL for vulpinic acid; 5–100 µg/mL for docetaxel). Throughout this manuscript, they will be referred to as lecanoric acid LA, evernic acid EA, and vulpinic acid VA.

### Cell viability assay

A549 cells were seeded into 96-well plates at a density of 7.5 × 10^3^ cells in 200 µL of culture medium and allowed to attach overnight at 37 °C in a 5% CO_2_ before being subjected to treatment. The seeded cells were incubated for 24 and 48 h after treatment with LA, EA, VA, and (positive control) docetaxel at various concentrations, and then their cytotoxic effects were assessed through an XTT assay (11465015001, Cell Proliferation Kit, Roche). In summary, the culture medium was removed, and 50 µL of XTT solution (consisting of 5 mL of XTT reagent and 1 mL of electron coupling reagent) was added to each well containing 100 µL of medium. The plates were then incubated at 37 °C with 5% CO_2_, and the absorbance at 470 nm was measured using a microplate spectrophotometer (Epoch Microplate Reader, BioTek, USA) (Günaydın et al. 2023). The results of cytotoxicity were expressed as IC_50_ (half maximal inhibitory concentration) values, and each value was accompanied by the standard deviation from triplicate experiments (± SD).

### Apoptosis assay

The impacts of lichen acids and hydrogen peroxide (H_2_O_2_) on the distribution of apoptotic and necrotic cell populations were assessed through the use of an Annexin V-fluorescein isothiocyanate (FITC)/propidium iodide (PI) double staining detection kit (640914, BioLegend, San Diego, CA). A549 cells were initially seeded in 6-well plates at a density of 3 × 10^5^ cells/well and incubated overnight at 37 °C. Subsequently, the cells were treated with EA and VA at their respective IC_50_ values for 24 and 48 h. After incubation, the cells were collected and washed twice with cold Dulbecco’s phosphate-buffered saline (D8662, DPBS, Sigma-Aldrich). The cells were then suspended in 100 µL of Annexin V-binding buffer (5 µL Annexin V-FITC and 10 µL PI) as per the manufacturer’s guidelines. After incubating the cells for 15 min at room temperature in the dark, 400 µL of Annexin V binding buffer was added to the samples. Finally, a Beckman Coulter CytoFLEX flow cytometer (Beckman Coulter, Brea, CA) was used to analyze the cell suspensions after they had been transferred to a 96-well plate (Altay et al. 2022). H_2_O_2_ was utilised as the positive control group, while untreated cells were used as the negative control group. The experimental procedures were carried out in triplicate.

### Wound healing assay

A549 cells (5 × 10^5^ cells/well) were seeded in a 6-well plate and allowed to attach by incubation in a 5% CO_2_ incubator at 37 °C until they reached 90% confluence. A 200 µL sterile pipette tip was used to scratch the center of the wells, and then DPBS was used to wash the cells. Following this, the cells were cultivated at 37 °C in fresh complete Ham’s F-12 K medium, with or without the addition of EA and VA. The wounds were photographed through an inverted microscope at 0, 6, 12, 24, and 48 h time points (Wang et al. 2018). The following formula was used to obtain the percentage rate of cell migration:

$$\mathrm{Percent}\;\mathrm{cell}\;\mathrm{migration}=(\mathrm{initial}\;\mathrm{scratch}\;\mathrm{width}-\mathrm{scratch}\;\mathrm{width}\;\mathrm{of}\;\mathrm{specified}\;\mathrm{time})/\mathrm{initial}\;\mathrm{scratch}\;\mathrm{width}\times100\%.$$

### cDNA synthesis and quantitative real-time PCR

Total RNA extractions from EA- and VA-treated A549 cells were performed by using RNA isolation PureLink™ RNA Mini Kit (12183018A, Invitrogen), and the purities and concentrations of the RNA were measured using a spectrophotometer (Thermo Fisher Scientific, Multiskan GO, USA). The High-Capacity cDNA Reverse Transcription Kit (4368814, Applied Biosystems) was utilised to synthesise first-strand cDNA in accordance with the manufacturer’s guidelines. The obtained cDNA was subsequently stored at −20 °C. The Rotor-Gene Q (Qiagen) instrument was utilised for quantitative real-time PCR (qPCR). In accordance with the protocol by the SYBR Green Master Mix (1,725,121, BioRad), qPCR analyses of BCL2 associated X, apoptosis regulator (BAX), BCL2 apoptosis regulator (BCL2), tumor protein p53 (P53), and TrxR1 target genes were conducted. The amplification of products was evaluated by amplification curve analysis. The gene expressions were conducted by using the 2^–∆∆CT^ method (Livak and Schmittgen 2001) and β-Actin was used as a housekeeping gene.

### Protein determination and western blotting analysis

A549 cells untreated (control group) and treated with EA and VA were harvested by scraping from cell culture petri dishes. The cells were then lysed in 400 µL of RIPA buffer (9806S, Cell Signaling Technology) containing 1 mM Phenylmethylsulfonyl fluoride (P7626, PMSF, Sigma-Aldrich). The spectrophotometric quantification of total protein amounts was carried out using the Bradford method (Bradford 1976) with the standard bovine serum albumin (A9647, BSA, Sigma-Aldrich). On a 12% SDS-PAGE gel, 40 µg of molecular weight protein was separated and then transferred to polyvinylidene fluoride (PVDF) membranes. Following blotting, the membranes were blocked for one hour at room temperature using 5% skim milk powder in TBST (Tris-buffered saline, 0.1% Tween 20) buffer. After blocking, the membranes were incubated at 4 °C for an overnight period with primary antibodies against β-Actin (sc-47778, Santa Cruz Biotechnology; 1:1000) and TrxR1 (sc-28321, Santa Cruz Biotechnology; 1:1000). Afterwards, the membranes were washed and incubated for 1 h at room temperature using horseradish‐coupled secondary antibodies (sc-2005, Santa Cruz Biotechnology; diluted 1:10,000). The enhanced chemiluminescent detection system (ECL Clarity/ECL Clarity Max Substrate, BioRad) was used to visualize the signals, and the pictures were analysed with the ImageJ2x program (Sulukoğlu et al. 2024).

### TrxR1 enzyme activity assay

The DTNB (5,5- dithiobis-2-nitrobenzoic acid) method, which is predicated on NADPH-dependent catalysis of the reduction of disulfide bonds in DTNB, was employed to measure the activity of the TrxR1 enzyme (Hill et al. 1997). The cell lysate was obtained by collecting control and treated A549 cells and lysing them in 400 μL RIPA buffer containing 1 mM PMSF was used. The test tube was filled with 200 μL K-phosphate buffer (100 mM, Potassium phosphate, Sigma-Aldrich) at pH 7/10 mM EDTA (ethylene diamine tetra acetic acid, Sigma-Aldrich) at pH 7, followed by 100 μL of BSA (0. 2 mg/mL, Sigma-Aldrich), 100 μL of DTNB (5 mM, Sigma-Aldrich), 100 μL of NADPH (0.2 mM, Sigma-Aldrich), 20 μL of supernatant and dH_2_O were added to obtain a final volume of 1000 μl. The spectrophotometric monitoring of TrxR1 enzyme activity was conducted every three minutes at a wavelength of 412 nm. TrxR1 activity is based on the detection of TNB (2-nitro-5-thiobenzoic acid) resulting from the reduction of DTNB per minute. The molar extinction coefficient for TNB was 14.15 M^(−1)^cm^(−1)^. An enzyme unit (EU) of TrxR1 activity was used as the generation of 1 micromolar of TNB per minute under these conditions. The specific activity of TrxR1 was expressed as EU/mg of protein (Sönmez Aydın et al. 2021; Hukkamlı et al. 2023).

### Statistical analysis

All experiments were obtained from three independent experiments. The statistical comparison of the results was conducted using GraphPad Prism Software version 8.0.2 for Windows (GraphPad Software, USA). For the XTT analysis, an unpaired t-test and two-way analysis of variance (ANOVA) following Tukey’s multiple comparison test were performed. A two-way ANOVA following Tukey’s multiple comparison test was used for flow cytometry. The qPCR, migration, western blotting, and enzyme activity analyses were employed using an unpaired t-test. The results were regarded as statistical differences at *p* > 0.05 = (ns = not significant), *p < 0.05 significant, **p < 0.01 very significant, ***p < 0.0001, and ****p < 0.0001 extremely significant.

## Results

### Cytotoxic activities of EA and VA on A549 cells

To assess the cytotoxic efficacy of lecanoric (LA), evernic (EA), and vulpinic (VA) acids on A549 cells, the cells were subjected to treatment with each aforementioned compound in a dose- and time-dependent manner using the XTT assay. LA was found to exert a slight cytotoxic effect on A549 cells at concentrations up to 250 μg/mL over a 24 h period. Even at this concentration, cell viability decreased by 34% compared to the control (Fig. 1B-C). EA demonstrated cytotoxic effects at ≥ 50 μg/mL at 24 h and ≥ 100 μg/mL at 48 h, with corresponding VA was cytotoxic to A549 cells at doses of at least 10 μg/mL in 24 h and at least > 25 μg/mL in 48 h. Calculations indicated that IC_50_ values for VA were 64.75 ± 2.05 μg/mL and 36.21 ± 0.94 μg/mL at 24 and 48 h, respectively. The IC_50_ value of docetaxel, a commercial drug widely used in chemotherapy, on A549 cells was determined as 49.93 ± 0.09 μg/mL at 24 h (Fig. 1B-C). As illustrated in the inverted light microscopy images, it was observed that the morphological shape of A549 cells treated with the highest dose of LA and IC_50_ concentrations (24 and 48 h) of EA and VA exhibited a notable change (Fig. 1D). The results indicated that 24 h and 48 h were the best IC_50_ values for EA and VA, respectively. However, the IC_50_ value of LA could not be calculated. Therefore, the effective IC_50_ values of EA and VA were employed in the subsequent stages of this study.

### Effects of EA and VA on apoptosis and necrosis in A549 cells

**Fig. 2:**
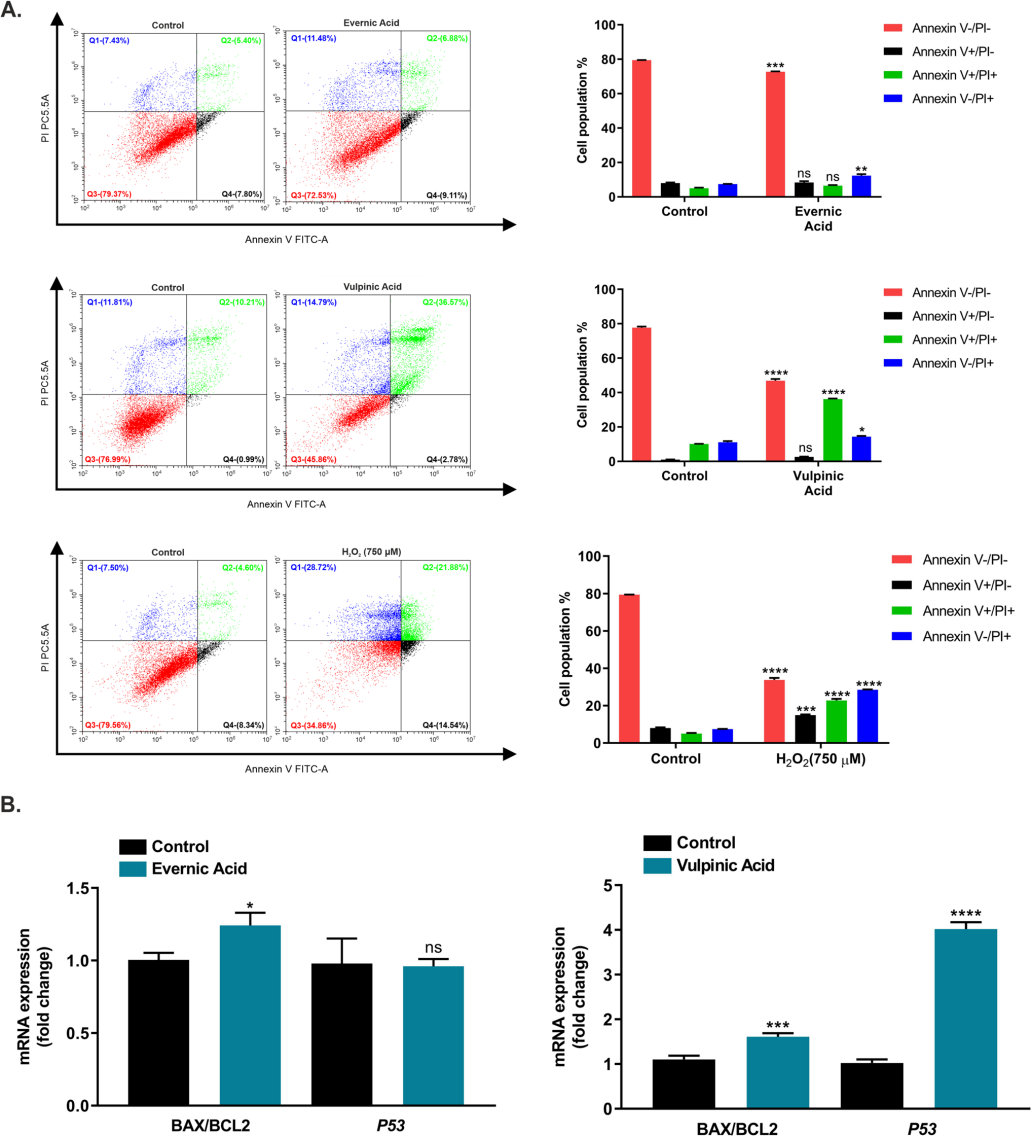
Apoptotic impacts of evernic and vulpinic acids in A549 cells. **A** A549 cells were treated with EA (IC_50_) for 24 h, and VA (IC_50_) for 48 h. Following this, the cells were double-stained with Annexin V-FITC/PI, and representative flow cytometry graphs were obtained. Hydrogen peroxide (H_2_O_2_, 750 µM) was used as a positive control. The four quadrants in the plot represent the four cell types: primary necrotic cells (Q1, Annexin V-/PI +), late apoptotic cells (Q2, Annexin V +/PI +), live cells (Q3, Annexin V-/PI-), and early apoptotic cells (Q4, Annexin V +/PI-). The percentage graphs of these cells are also shown. **B **The impacts of EA and VA on the quantitative gene expression of the apoptotic pathway (BAX/BCL2 ratio and the *P53*) were evaluated using qPCR. The graphical data are expressed as the mean ± S.E.M. of three independent experiments. *p* > 0.05 = (ns = not significant), *p < 0.05 significant, **p < 0.01 very significant, ***p < 0.0005 and ****p < 0.0001 extremely significant

To ascertain the apoptotic and necrotic effects of EA and VA, A549 cells were treated with the effective IC_50_ values of the test compounds and analysed using flow cytometry. The results unveiled that EA and VA reduced viable cell percentages by 6.73 ± 0.42% and 30.82 ± 0.43%, respectively, in comparison to the negative control. The administration of EA resulted in an elevation in the percentage of total apoptotic cells (Annexin V +/PI- and Annexin V +/PI +) by 1.84 ± 1.71% and necrotic cells by 4.89 ± 1.29%. The other test compound, VA, demonstrated a statistically significant increase in the percentages of total apoptotic cells, and necrotic cells by 27.59 ± 0.78%, and 3.23 ± 0.35%, respectively, compared to the control cells. On the other hand, H_2_O_2_ (used as positive control) at a high dose of 750 µM decreased the percentage of viable cells by 45.69 ± 1.39% and increased the percentage of total apoptotic cells by 24.61 ± 1.59%, necrotic cells by 21.07 ± 0.21%, compared to the control cells (Fig. 2A).

Furthermore, to figure out the apoptotic effects of EA and VA in A549 cells at the molecular level, quantitative mRNA expressions of *BAX*, *BCL2*, and *P53* were investigated using qPCR. For this, A549 cells were treated with the effective IC_50_ values of EA and VA, and the obtained data was compared to the control group. EA was observed to elevate the BAX/BCL2 ratio (*p* < 0.05), yet no notable change was determined in *P53* expression (*p* > 0.05). VA was found to increase both the BAX/BCL2 ratio considerably (*p* < 0.0005) and the relative mRNA expression of *P53* (*p* < 0.0001) (Fig. 2B).

### The antimigratory effects of EA and VA on A549 cells

**Fig. 3:**
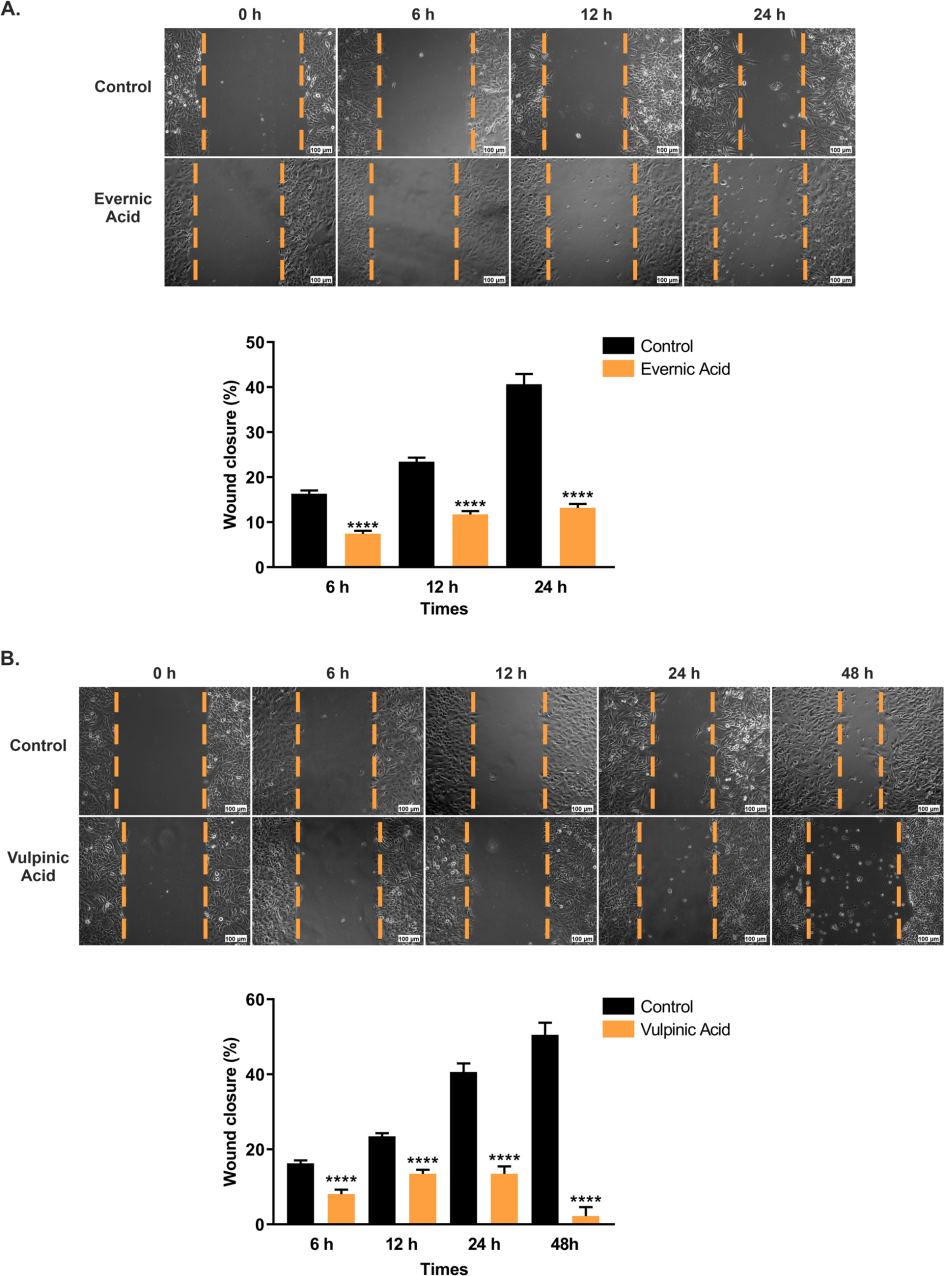
Antimigrative effects of evernic and vulpinic acids in A549 cells. Inverted light microscope images were obtained at 0, 6, 12, 24, and 48 h after exposure of A549 cells to the IC_50_ values of (**A**) EA and (**B**) VA using the wound healing assay. The dashed orange line represents the extent of the wounds in A549 cells. The percentage of wound closure was calculated by measuring the rate of cell migration at 6, 12, 24, and 48 h using the ImageJ software. The graphical data are expressed as the mean ± S.E.M. of data from four measurements of each wounded area, taken from three independent experiments (*n* = 12). ****p < 0.0001 extremely significant. Scale bar: 100 μm

A wound-healing assay was conducted in order to ascertain the impact of EA and VA on the migration of A549 cells. For this, scratches were created on the cells, which were subsequently treated with effective IC_50_ concentrations of EA and VA. Microscopic images were acquired at different times (0, 6, 12, 24, and 48 h) to observe the effect of the treatment. The data demonstrated that cells in the control group migrated rapidly to the wound area when compared to those treated with EA and VA. The statistical results indicated that EA significantly suppressed the migration of A549 cells by 8.85% at 6 h, 11.74% at 12 h, and 27.48% at 24 h in comparison to the control group (*p* < 0.0001) (Fig. 3A). VA exhibited a remarkable inhibitory effect on cell migration, with a reduction of 8.21% at 6 h, 9.96% at 12 h, 27.16% at 24 h, and 48.3% at 48 h compared to the untreated cells (*p* < 0.0001) (Fig. 3B).

### The impacts of EA and VA on TrxR1 protein expression and enzyme activity in A549 cells

**Fig. 4:**
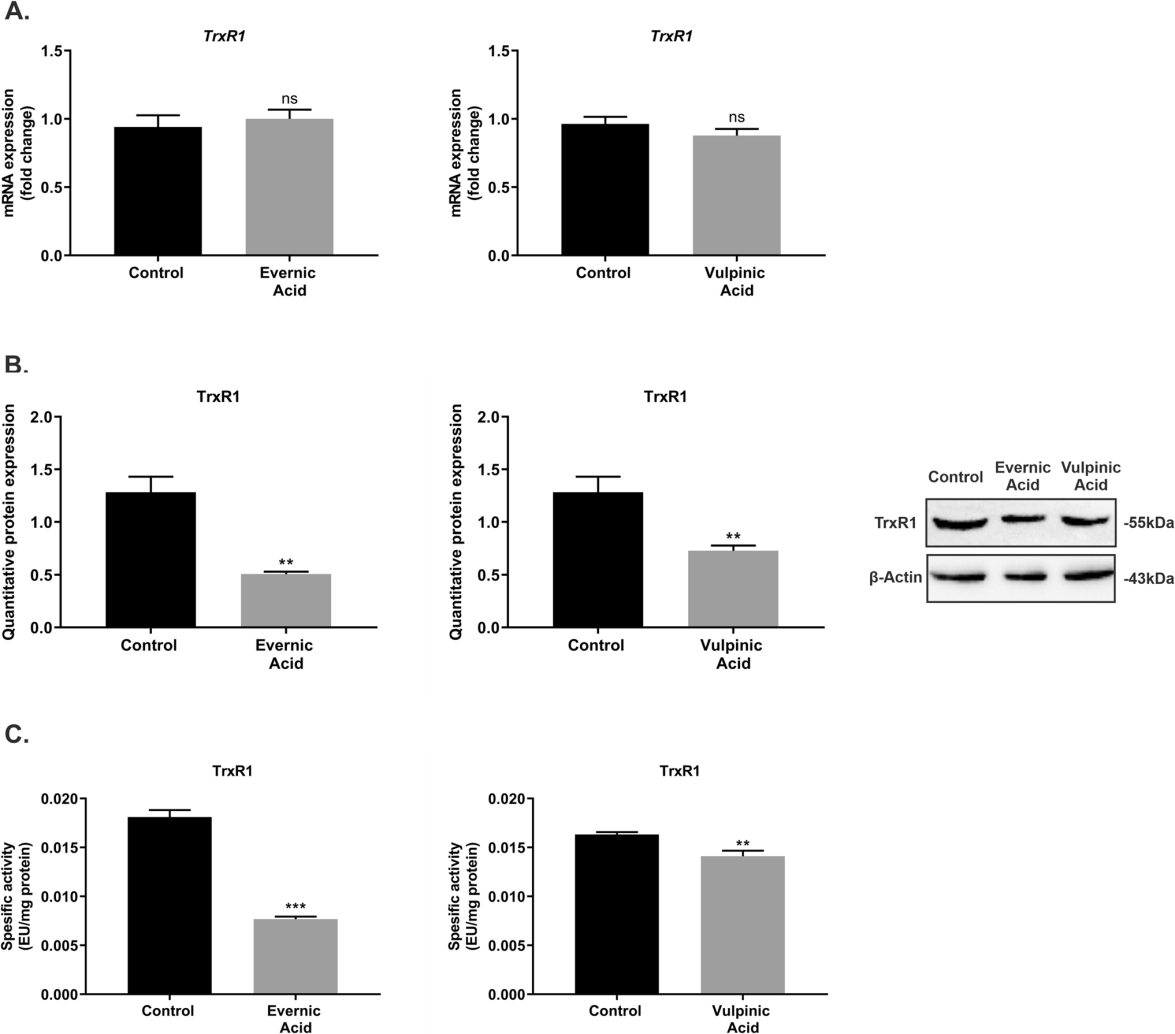
Effects of evernic and vulpinic acids on TrxR1 in A549 cells at the gene and protein levels. After A549 cells were exposed to the IC_50_ values of the EA and VA, quantitative mRNA expressions (**A**), quantitative protein expressions (**B**), and specific enzyme activities (**C**) of TrxR1 were analysed using qPCR, western blot, and the DTNB method, respectively. The graphical data are expressed as the mean ± S.E.M. of three independent experiments. *p* > 0.05 = (ns = not significant), **p < 0.01 very significant, and ***p < 0.0005 extremely significant

The expression of TrxR1 in cancer cells is markedly increased, and its potential as a therapeutic target in cancer treatment is being investigated. To this end, the TrxR1-related anticancer effects of EA and VA on A549 cells were evaluated at the gene, protein, and enzymatic levels, employing qPCR, western blot, and enzymatic activity studies. The qPCR results demonstrated that EA and VA had no notable impact on the mRNA and protein expression levels of TrxR1 in A549 cells when compared to the control cells (*p* > 0.05) (Fig. 4A). The protein expression of TrxR1 in A549 cells was significantly decreased (*p* < 0.01) by EA and VA (Fig. 4B). Additionally, EA (*p* < 0.0001) and VA (*p* < 0.01) demonstrated a notable inhibitory impact on the specific enzyme activity of TrxR1 (Fig. 4C).

## Dıscussıon

Lung cancer, which ranks first among cancer-related deaths, is a serious disease that is difficult to diagnose and treat. The application of diverse therapeutic modalities, including chemotherapy, is contingent upon the specific type, stage, and extent of the disease. However, chemotherapy is associated with several limitations, including the emergence of chemoresistance and the manifestation of adverse effects (Chang 2011; Testa et al. 2018). For this reason, there is a need to identify new chemotherapeutic agents possessing target-specific, minimal side effects, and natural product-based (Cragg and Pezzuto 2016; Castañeda et al. 2022). The present study was designed to investigate the anticancer potential of lecanoric (LA), evernic (EA), and vulpinic (VA) acids, which are secondary metabolites of lichen, on A549 cells. Additionally, the study aimed to ascertain whether this potential effect is occuring by targeting TrxR1.

To determine the antiproliferative effects of LA, EA, and VA on A549 cells, an initial examination was conducted using the XTT assay. As illustrated in Fig. 1, LA exhibited the least cytotoxic activity in comparison to the other lichen acids. EA demonstrated an effective IC₅₀ dose of 139.09 µg/mL at 24 h, whereas VA exhibited an effective IC₅₀ dose of 36.21 µg/mL at 48 h. VA was observed to have a more potent cytotoxic effect with a lower IC₅₀ value compared to EA and docetaxel.

In determining the cytotoxicity of a compound, the chemical structure of the scaffold plays a crucial role. Scaffolds are the core structures or skeletons of molecules, often serving as the basis for biological functionalization (Aishwarya et al. 2025). Aromatic hydrocarbons or heterocyclic systems (e.g. anthraquinones, indoles) can intercalate into DNA or interact with proteins, resulting in cytotoxic effects (Karthikeyan et al. 2023). For instance, doxorubicin with planar aromatic systems shows cytotoxicity in cancer cells by means of DNA intercalation and ROS generation (Khan et al. 2024). An electron donating group (EDG) such as hydroxyl (•OH) and methoxy (•OCH₃) on an aromatic ring can improve a drug’s ability to interact with its target, increasing cytotoxicity or activity in cancer cells (Venkatesan et al. 2024). However, in some cases, transferring too many electrons can make some compounds too reactive or unstable to be effective. (Halliwell 2024). Looking at the chemical structures of LA and EA tested in this study, it can be seen that the scaffold of both compounds are the same. LA contains •OH as an electron donor to the aromatic ring while EA contains •OCH_3_, and this is the only structural difference. Although LA was expected to be more prominent in terms of cytotoxicity, EA was found to have stronger cytotoxic activity against A549 cells. This may be due to the high reactivity and instability of lecanoric acid in the cells. Another important point is that substitution of •OH on aromatic scaffolds increases antioxidant activity and suppresses cytotoxicity to some extent (Kubiak-Tomaszewska et al. 2022).

The ester groups are often lipophilic, which makes it easier for the compounds to pass through the cell membrane. This property can have a positive effect on the bioavailability of cytotoxic drugs (Keydel and Link 2024). To improve solubility or membrane permeability, many cytotoxic drugs are designed as prodrugs with ester groups and enzymatic cleavage of the ester group in the target cell releases the active cytotoxic agent (Nazli et al. 2024). Looking at the structure of VA, it contains a lactone derivative with an extra cyclic ester between two aromatic rings, making it slightly more lipophilic than LA and EA. According to XTT analysis, the fact that VA is more cytotoxic than other acids may be due to its cell membrane permeability, resulting in greater ROS production and enzyme inhibition within the cell.

In the literature, EA was stated to reduce the proliferation of A549 cells in a concentration range of 12.5–100 µg/mL, without determining the IC_50_ value (Kizil et al. 2015). In a study by Studzińska-Sroka et al., it was reported that EA has a moderate cytotoxic effect in the concentration range of 10–100 μM in human glioblastoma cell lines A-172 and at only 100 μM in T98G (Studzińska-Sroka et al. 2021). Roser et al. showed that EA did not affect cell viability in HCT-116 (colon cancer epithelial cell line), HEK293T (human embryonal kidney), RAW246.7 (murine leukemic macrophage), and HeLa (human cervical epithelial cancer) cell lines at concentrations up to 30 µg/mL at 24 h. They also reported that only 30 µg/mL decreased the viability of NIH3T3 (murine fibroblast) cells. It was reported that LA exhibited a reduction in cell viability at concentrations of 0.3 µg/mL for HEK293, 3 µg/mL for HeLa, NIH3T3, and RAW264.7, and 30 µg/mL for HCT-116 (Roser et al. 2022). In another study, it was demonstrated that LA exhibited an IC_50_ value exceeding 50 µg/mL in breast carcinoma (MCF-7), larynx carcinoma (Hep-2), and normal (Vero) cell lines. In addition, LA derivatives have been reported to increase cytotoxic activity (Bogo et al. 2010).

It has been documented in the literature that VA exhibits antiproliferative activity against a multitude of cancer cells, including malignant mesothelioma, vulvar carcinoma, keratinocyte cells (Burlando et al. 2009), colorectal adenocarcinoma, hepatocellular carcinoma, cervical carcinoma, rhabdomyosarcoma, and mouse fibrosarcoma (Sahin et al. 2019), breast cancer (Kılıc et al. 2018; Kalın et al. 2022a), as well as cervical cancer (Budak et al. 2024). In the study conducted by (Kim et al. 2017), it was stated that VA, which was determined to be the primary component of the hexane fraction of the methanol (MeOH) extract of *Pulveroboletus ravenelii*, exhibited a notable reduction in the viability of lung, pancreatic ductal adenocarcinoma, and hepatocellular carcinoma cells. (Burlando et al. 2009) reported that EA exhibited low cytotoxicity, while VA demonstrated moderate cytotoxicity. EA was applied at concentrations up to 200 µg/mL to healthy human HUVEC cells, and no significant decrease in cell proliferation was observed (Kizil et al. 2015). A review of the literature reveals a esearch investigating the effect of EA on ovarian cancer cell lines and normal ovarian surface epithelial cells (OSE) cells. The findings indicate that the substance reduces cell proliferation in ovarian cancer cell lines at IC_50_ concentrations ranging from 10 to 65.4 μM over a period of 60 to 68 h. Furthermore, EA was found to reduce the proliferation of normal cells at an IC_50_ concentration of 159.5 μM (approximately 53.0 μg/mL) at OSE d 97 h. They reported that EA is a promising potential therapeutic agent for ovarian cancer without showing significant cytotoxic effects on normal cells (Ensoy et al. 2025). VA has been demonstrated to decrease cell viability in healthy human umbilical vein endothelial cells (HUVECs) at IC_50_ concentrations of 1890.51 ± 3.56 μM (approximately 609.3 μg/mL) after 24 h and 231.94 ± 25.4 μM (approximately 74.75 μg/mL) after 48 h. Thus, it was demonstrated that VA exhibited greater cytotoxicity towards cancer cells while displaying reduced cytotoxicity towards the healthy HUVEC cell line (Koparal 2015). The study highlighted that VA demonstrated potent antiproliferative efficacy against lung cancer cell lines (SPC-A-1, 95D and SK-LU-1), exhibiting a stronger inhibitory effect than the chemotherapeutic drug doxorubicin (Wu et al. 2022). Our results are consistent with the literature, which suggests that the discrepancies in the reported cytotoxicity of lichen acids are likely due to the varying degrees of purity of the metabolites produced by the different isolation techniques, as well as the types of cancer cell lines and cytotoxicity methods. When the data obtained from this study for EA and VA are compared with the literature, it is an important finding that they showed cytotoxic effects on A549 lung cancer cells without causing substantial toxicity to healthy cells. This finding lends support to the notion that EA and VA have the potential to function as a targeted therapeutic agent in cancerous cells.

The two main forms of cell death mechanisms are apoptosis and necrosis, both of which play a key role in the discovery of anticancer drugs in various physiological and pathological conditions, especially in the elimination of cancer cells (Lekshmi et al. 2017). The interaction between all BCL2 family members controls the balance of apoptotic and necrotic cell death in response to various stimuli. In the process of apoptosis, BAX and BAK oligomerize in the outer mitochondrial membrane, release of substrates that trigger apoptosis, and the subsequent activation of caspases and nucleases (Karch and Molkentin 2015). The BAX/BCL2 ratio, which determines the susceptibility of cells to apoptosis, can affect cancer cell behavior, and its high levels lead cells to apoptosis (Khodapasand et al. 2015). Conversely, in necrosis, an uncontrolled form of cell death, mitochondria become dysfunctional and inadaptive, with the production of ROS and loss of ATP production, in part through the opening of the mitochondrial permeability transition pore. Recent studies have demonstrated that necrosis can be a highly regulated form of cell death in adult vertebrates under certain cellular contexts. Although regulated necrosis is caspase-independent, recent evidence has demonstrated that it nevertheless requires the apoptotic regulators BAX and BAK, which can regulate the permeability properties of the outer mitochondrial membrane in their non-oligomerized state (Karch and Molkentin 2015). The induction of apoptosis and/or necrosis by the use of natural compounds also represents a new focus and offers insights into anticancer mechanisms (Wei et al. 2020; Yu et al. 2020).

To date, only a limited number of studies have been conducted on the apoptotic effect of EA and VA. This study aimed to investigate the effects of EA and VA on the expression of apoptotic marker genes *BAX*, *BCL2*, and *P53* in A549 cells. The findings revealed that EA and VA elevated the BAX/BCL2 ratio, but only VA increased the *P53* expression. The results of the flow cytometric analysis indicated that VA promoted apoptosis and necrosis in A549 cells, whereas EA induced a relatively low rate of these events. These results are consistent with the findings reported by Roser, which indicated that EA did not exhibit an apoptotic effect on HCT116 cells (Roser et al. 2022). Regarding the apoptotic data obtained with VA, it was found to be consistent with those reported. For instance, VA, which had previously been reported to be the cytotoxic component of *Pulveroboletus ravenelii*, was demonstrated to induce apoptosis in lung, pancreatic, and hepatocellular cancer cells. However, it was emphasised that the underlying mechanisms for its cytotoxic potential require further investigation (Kim et al. 2017). A study reported that VA upregulated *BAX* and *P53* levels in Caco-2, HepG2, Hep2C, RD, and Wehi cancer cells and had a role in apoptosis (Sahin et al. 2019). Furthermore, it has been indicated that VA induces apoptosis in breast, prostate, and melanoma cancer cells (Cansaran-Duman et al. 2021a, b; Yangın et al. 2022). Overall, these findings suggest that VA is more effective in promoting apoptosis and necrosis of A549 cells.

Metastasis, which is closely connected to many cancer-related deaths, i.e. lung cancer, is one of the most important events to be focused on cancer treatment and prevention (Wood et al. 2014; Qian et al. 2017). In the study examining the effect of lichen acids on the migration of HaCaT cells, it was reported that EA did not make any difference in wound closure rate, while VA showed a moderate effect (Burlando et al. 2009). In the current study, we examined the effect of EA and VA on the migration of A549 cells. The present study exhibited that EA and VA strongly suppressed the migration of A549 cells, suggesting that further investigation into the metastatic cascade of both lichen acids is warranted.

Cancer cells adapt to oxidative stress by increasing the expression of various antioxidant enzymes and proteins to a greater extent than is observed in normal cells. A review of the literature reveals that numerous pathways associated with tumourigenesis regulate ROS directly or indirectly (Rajavel et al. 2019). TrxR1, which plays a pivotal role in providing redox balance, functions as a double-edged sword. In normal cells, TrxR1 performs a number of functions, including the regulation of apoptosis and the protection of cells against oxidative stress. Nevertheless, overexpression of TrxR1 is closely associated with cancer pathology, including cell proliferation and metastasis. Consequently, TrxR1-targeted inhibition has been accepted as a rational therapeutic approach for cancer treatment (Mahmood et al. 2013; Arnér 2017; Gencheva and Arnér 2022).

The characterisation of target proteins in cancer cells is a noteworthy area of research in the development of novel and specific chemotherapeutic agents (Zhang et al. 2017; Das Mukherjee et al. 2020). Natural products, such as lichen secondary metabolites, are recognised as one of the most plausible strategies for the discovery of new anticancer agents (Bačkorová et al. 2012; Kalın et al. 2022b, 2023; Günaydın et al. 2023; Sulukoğlu et al. 2024). In this regard, we purposed to examine whether the anticancer effect of EA and VA is associated with the inhibition of TrxR1 in A549 cells. The findings of our present study revealed that EA and VA did not cause a significant change in the gene expression level of TrxR1 in A549 cells. However, these substances caused to a notable reduction in the protein expression levels of TrxR1 in A549 cells, accompanied by a substantial inhibition of enzyme activity. This difference in gene and protein expression levels suggests that there is a low to moderate correlation between gene and protein expressions in the literature and that the effect is shown at the protein level rather than the gene level (Meiners et al. 2003; Cheng et al. 2016). In the literature, the inhibitory effect of EA and VA on mitochondrial TrxR isolated from rat lung was shown to be significantly stronger than that of the commercial anticancer medications doxorubicin and cisplatin (Ozgencli et al. 2019). The findings of this study demonstrated that the existing lichen acids exhibited anticancer effects through the thioredoxin system. However, it is acknowledged that the glutathione antioxidant defence system is also present in cells in addition to the thioredoxin system. The system is composed of the following primary components: GPX, GR, SOD and CAT. Consequently, more comprehensive comments can be made following the investigation of the effects of EA and VA on these enzymes in future studies.

## Conclusion

In summary, VA has a more potent cytotoxic effect than EA and the anticancer drug docetaxel, but lecanoric acid has no cytotoxic activity. EA and VA exhibited cytotoxic, necrotic, and anti-migratory activity on A549 cells, whereas only VA exhibited apoptotic activity. EA and VA, which have no effect at the gene level, suppressed TrxR1 protein expression and inhibited enzyme activity. These findings suggest that evernic acid and vulpinic acid could act as TrxR1 inhibitors and new potential chemotherapeutic agents for lung cancer.

## Data Availability

All source data for this work (or generated in this study) are available upon reasonable request.

## Abbreviations

A549: Human lung cancer cell line
BAX: BCL2 associated X, apoptosis regulator
BCL2: BCL2 apoptosis regulator
DMSO: Dimethyl Sulfoxide
DPBS: Dulbecco’s Phosphate-Buffered Saline
EA: Evernic Acid
Ham’s F-12K: Ham’s F-12 K (Kaighn’s) medium
LA: Lecanoric acid
NSCLC: Non-small cell lung cancer
P53: Tumor protein p53
qPCR: Quantitative real-time PCR
ROS: Reactive oxygen species
TrxR1: Thioredoxin reductase 1
VA: Vulpinic Acid

## References

[CR1] Aishwarya NVSS, Matada GSP, Pal R et al (2025) Expanding the potential of pyridine scaffold for targeted therapy of cancer: Biological activity, molecular insights, and structure-activity relationship. J Mol Struct 1321:139655. 10.1016/j.molstruc.2024.139655

[CR2] Altay A, Caglar S, Caglar B (2022) Silver(I) complexes containing diclofenac and niflumic acid induce apoptosis in human-derived cancer cell lines. Arch Physiol Biochem 128:69–79. 10.1080/13813455.2019.1662454 31516039 10.1080/13813455.2019.1662454

[CR3] Arnér ESJ (2017) Targeting the selenoprotein thioredoxin reductase 1 for anticancer therapy. In: Advances in cancer research. Academic Press, pp 139–151 10.1016/bs.acr.2017.07.005 29054416

[CR4] Bačkorová M, Jendželovský R, Kello M et al (2012) Lichen secondary metabolites are responsible for induction of apoptosis in HT-29 and A2780 human cancer cell lines. Toxicol Vitr 26:462–468. 10.1016/j.tiv.2012.01.017 10.1016/j.tiv.2012.01.017 22285236

[CR5] Belhouala K, Korkmaz C, Taş Küçükaydın M et al (2024) Eco-friendly species Evernia prunastri (L.) Ach.: phenolic profile, antioxidant, anti-inflammatory, and anticancer properties. ACS Omega 9:45719–45732. 10.1021/acsomega.3c10407 39583657 10.1021/acsomega.3c10407 PMC11579742

[CR6] Bogo D, Matos MDFC, Honda NK et al (2010) In vitro antitumour activity of orsellinates. Z Naturforsch C 65:43–48. 10.1515/znc-2010-1-208 20355320 10.1515/znc-2010-1-208

[CR7] Bradford M (1976) A rapid and sensitive method for the quantitation of microgram quantities of protein utilizing the principle of protein-dye binding. Anal Biochem 72:248–254. 10.1006/abio.1976.9999 942051 10.1016/0003-2697(76)90527-3

[CR8] Budak B, Kalın ŞN, Yapça ÖE (2024) Antiproliferative, antimigratory, and apoptotic effects of diffractaic and vulpinic acids as thioredoxin reductase 1 inhibitors on cervical cancer. Naunyn Schmiedebergs Arch Pharmacol 397:1525–1535. 10.1007/s00210-023-02698-w 37658214 10.1007/s00210-023-02698-w

[CR9] Burlando B, Ranzato E, Volante A et al (2009) Antiproliferative effects on tumour cells and promotion of keratinocyte wound healing by different lichen compounds. Planta Med 75:607–613. 10.1055/s-0029-1185329 19199230 10.1055/s-0029-1185329

[CR10] Cansaran-Duman D, Guney Eskiler G, Colak B, Sozen Kucukkara E (2021a) Vulpinic acid as a natural compound inhibits the proliferation of metastatic prostate cancer cells by inducing apoptosis. Mol Biol Rep 48:6025–6034. 10.1007/s11033-021-06605-5 34331181 10.1007/s11033-021-06605-5

[CR11] Cansaran-Duman D, Yangın S, Çolak B (2021b) The role of vulpinic acid as a natural compound in the regulation of breast cancer-associated miRNAs. Biol Res 54:37. 10.1186/s40659-021-00360-4 34743742 10.1186/s40659-021-00360-4 PMC8574026

[CR12] Castañeda AM, Meléndez CM, Uribe D, Pedroza-Díaz J (2022) Synergistic effects of natural compounds and conventional chemotherapeutic agents: recent insights for the development of cancer treatment strategies. Heliyon 8:e09519. 10.1016/j.heliyon.2022.e09519 35669542 10.1016/j.heliyon.2022.e09519 PMC9163513

[CR13] Chaitanya Thandra K, Barsouk A, Saginala K et al (2021) Epidemiology of lung cancer. Współczesna Onkol 25:45–52. 10.5114/wo.2021.103829 10.5114/wo.2021.103829 PMC8063897 33911981

[CR14] Chang A (2011) Chemotherapy, chemoresistance and the changing treatment landscape for NSCLC. Lung Cancer 71:3–10. 10.1016/j.lungcan.2010.08.022 20951465 10.1016/j.lungcan.2010.08.022

[CR15] Cheng Z, Teo G, Krueger S, et al (2016) Differential dynamics of the mammalian mRNA and protein expression response to misfolding stress. Mol Syst Biol 12. 10.15252/msb.20156423 10.15252/msb.20156423 PMC4731011 26792871

[CR16] Cragg GM, Pezzuto JM (2016) Natural products as a vital source for the discovery of cancer chemotherapeutic and chemopreventive agents. Med Princ Pract 25:41–59. 10.1159/000443404 26679767 10.1159/000443404 PMC5588531

[CR17] Das Mukherjee D, Kumar NM, Tantak MP et al (2020) NMK-BH2, a novel microtubule-depolymerising bis (indolyl)-hydrazide-hydrazone, induces apoptotic and autophagic cell death in cervical cancer cells by binding to tubulin at colchicine – site. Biochim Biophys Acta - Mol Cell Res 1867:118762. 10.1016/j.bbamcr.2020.118762 32502617 10.1016/j.bbamcr.2020.118762

[CR18] Ebrahim HY, Elsayed HE, Mohyeldin MM et al (2016) Norstictic acid inhibits breast cancer cell proliferation, migration, invasion, and in vivo invasive growth through targeting C-Met. Phyther Res 30:557–566. 10.1002/ptr.5551 10.1002/ptr.5551 PMC5045260 26744260

[CR19] Ensoy M, Parıltı DN, Alkan AH, et al (2025) Evernic acid: a low‐toxic and selective alternative to chemotherapeutic agents in the treatment of ovarian cancer. Arch Pharm (Weinheim) 358. 10.1002/ardp.70015 10.1002/ardp.70015 PMC12099196 40405479

[CR20] Fan C, Zheng W, Fu X et al (2014) Enhancement of auranofin-induced lung cancer cell apoptosis by selenocystine, a natural inhibitor of TrxR1 in vitro and in vivo. Cell Death Dis 5:e1191–e1191. 10.1038/cddis.2014.132 24763048 10.1038/cddis.2014.132 PMC4001298

[CR21] Gencheva R, Arnér ESJ (2022) Thioredoxin reductase inhibition for cancer therapy. Annu Rev Pharmacol Toxicol 62:177–196. 10.1146/annurev-pharmtox-052220-102509 34449246 10.1146/annurev-pharmtox-052220-102509

[CR22] Günaydın Ş, Sulukoğlu EK, Kalın ŞN et al (2023) Diffractaic acid exhibits thioredoxin reductase 1 inhibition in lung cancer A549 cells. J Appl Toxicol 43:1676–1685. 10.1002/jat.4505 37329199 10.1002/jat.4505

[CR23] Halliwell B (2024) Understanding mechanisms of antioxidant action in health and disease. Nat Rev Mol Cell Biol 25:13–33. 10.1038/s41580-023-00645-4 37714962 10.1038/s41580-023-00645-4

[CR24] Hill KE, McCollum GW, Burk RF (1997) Determination of thioredoxin reductase activity in rat liver supernatant. Anal Biochem 253:123–125. 10.1006/abio.1997.2373 9356150 10.1006/abio.1997.2373

[CR25] Hukkamlı B, Dağdelen B, Sönmez Aydın F, Budak H (2023) Comparison of the efficacy of the mouse hepatic and renal antioxidant systems against inflammation-induced oxidative stress. Cell Biochem Biophys. 10.1007/s12013-023-01126-3 36773183 10.1007/s12013-023-01126-3

[CR26] Jovanović M, Dragoj M, Zhukovsky D, et al (2020) Novel TrxR1 inhibitors show potential for glioma treatment by suppressing the invasion and sensitizing glioma cells to chemotherapy. Front Mol Biosci 7. 10.3389/fmolb.2020.586146 10.3389/fmolb.2020.586146 PMC7573255 33134322

[CR27] Kalın ŞN, Altay A, Budak H (2022a) Inhibition of thioredoxin reductase 1 by vulpinic acid suppresses the proliferation and migration of human breast carcinoma. Life Sci 310:121093. 10.1016/j.lfs.2022.121093 36270425 10.1016/j.lfs.2022.121093

[CR28] Kalın ŞN, Altay A, Budak H (2022b) Diffractaic acid, a novel TrxR1 inhibitor, induces cytotoxicity, apoptosis, and antimigration in human breast cancer cells. Chem Biol Interact 361:109984. 10.1016/j.cbi.2022.109984 35569514 10.1016/j.cbi.2022.109984

[CR29] Kalın ŞN, Altay A, Budak H (2023) Effect of evernic acid on human breast cancer MCF-7 and MDA-MB-453 cell lines via thioredoxin reductase 1: a molecular approach. J Appl Toxicol. 10.1002/jat.4451 36807289 10.1002/jat.4451

[CR30] Karch J, Molkentin JD (2015) Regulated necrotic cell death. Circ Res 116:1800–1809. 10.1161/CIRCRESAHA.116.305421 25999420 10.1161/CIRCRESAHA.116.305421 PMC4443748

[CR31] Karthikeyan S, Grishina M, Kandasamy S et al (2023) A review on medicinally important heterocyclic compounds and importance of biophysical approach of underlying the insight mechanism in biological environment. J Biomol Struct Dyn 41:14599–14619. 10.1080/07391102.2023.2187640 36914255 10.1080/07391102.2023.2187640

[CR32] Keydel T, Link A (2024) Synthetic approaches, properties, and applications of acylals in preparative and medicinal chemistry. Molecules 29:4451. 10.3390/molecules29184451 39339447 10.3390/molecules29184451 PMC11434492

[CR33] Khan HY, Ansari MF, Tabassum S, Arjmand F (2024) A review on the recent advances of interaction studies of anticancer metal-based drugs with therapeutic targets, DNA and RNAs. Drug Discov Today 29:104055. 10.1016/j.drudis.2024.104055 38852835 10.1016/j.drudis.2024.104055

[CR34] Khodapasand E, Jafarzadeh N, Farrokhi F et al (2015) Is Bax/Bcl-2 ratio considered as a prognostic marker with age and tumor location in colorectal cancer? Iran Biomed J 19:69–75. 10.6091/ibj.1366.2015 25864810 10.6091/ibj.1366.2015 PMC4412916

[CR35] Kim S, So HM, Roh H-S et al (2017) Vulpinic acid contributes to the cytotoxicity of Pulveroboletus ravenelii to human cancer cells by inducing apoptosis. RSC Adv 7:35297–35304. 10.1039/C7RA05059C

[CR36] Kizil HE, Agar G, Anar M (2015) Antiproliferative effects of Evernic acid on A549 and healthy human cells: an in vitro study. J Biotechnol 208:S28. 10.1016/j.jbiotec.2015.06.074

[CR37] Kılıc N, Derici MK, Buyuk I et al (2018) Evaluation of in vitro anticancer activity of vulpinic acid and its apoptotic potential using gene expression and protein analysis. Indian J Pharm Educ Res 52:626–634. 10.5530/ijper.52.4.73

[CR38] Koparal AT (2015) Anti-angiogenic and antiproliferative properties of the lichen substances (-)-usnic acid and vulpinic acid. Z Naturforsch C 70:159–164. 10.1515/znc-2014-4178 26136299 10.1515/znc-2014-4178

[CR39] Kosanić M, Manojlović N, Janković S et al (2013) Evernia prunastri and Pseudoevernia furfuraceae lichens and their major metabolites as antioxidant, antimicrobial and anticancer agents. Food Chem Toxicol 53:112–118. 10.1016/j.fct.2012.11.034 23220145 10.1016/j.fct.2012.11.034

[CR40] Kubiak-Tomaszewska G, Roszkowski P, Grosicka-Maciąg E et al (2022) Effect of hydroxyl groups esterification with fatty acids on the cytotoxicity and antioxidant activity of flavones. Molecules 27:420. 10.3390/molecules27020420 35056733 10.3390/molecules27020420 PMC8777613

[CR41] Lekshmi A, Varadarajan SN, Lupitha SS et al (2017) A quantitative real-time approach for discriminating apoptosis and necrosis. Cell Death Discov 3:16101. 10.1038/cddiscovery.2016.101 28179996 10.1038/cddiscovery.2016.101 PMC5253725

[CR42] Lemjabbar-Alaoui H, Hassan OU, Yang Y-W, Buchanan P (2015) Lung cancer: biology and treatment options. Biochim Biophys Acta - Rev Cancer 1856:189–210. 10.1016/j.bbcan.2015.08.002 10.1016/j.bbcan.2015.08.002 PMC4663145 26297204

[CR43] Li Y, Yan B, He S (2023) Advances and challenges in the treatment of lung cancer. Biomed Pharmacother 169:115891. 10.1016/j.biopha.2023.115891 37979378 10.1016/j.biopha.2023.115891

[CR44] Livak KJ, Schmittgen TD (2001) Analysis of relative gene expression data using real-time quantitative PCR and the 2−ΔΔCT method. Methods 25:402–408. 10.1006/meth.2001.1262 11846609 10.1006/meth.2001.1262

[CR45] Luo H, Yamamoto Y, Kim AJ et al (2009) Lecanoric acid, a secondary lichen substance with antioxidant properties from Umbilicaria antarctica in maritime Antarctica (King George Island). Polar Biol 32:1033–1040. 10.1007/s00300-009-0602-9

[CR46] Mahmood DFD, Abderrazak A, El Hadri K et al (2013) The thioredoxin system as a therapeutic target in human health and disease. Antioxid Redox Signal 19:1266–1303. 10.1089/ars.2012.4757 23244617 10.1089/ars.2012.4757

[CR47] Meiners S, Heyken D, Weller A et al (2003) Inhibition of proteasome activity induces concerted expression of proteasome genes and de novo formation of mammalian proteasomes. J Biol Chem 278:21517–21525. 10.1074/jbc.M301032200 12676932 10.1074/jbc.M301032200

[CR48] Molnár K, Farkas E (2010) Current results on biological activities of lichen secondary metabolites: a review. Z Naturforsch C 65:157–173. 10.1515/znc-2010-3-401 20469633 10.1515/znc-2010-3-401

[CR49] Nazli A, Irshad Khan MZ, Rácz Á, Béni S (2024) Acid-sensitive prodrugs; a promising approach for site-specific and targeted drug release. Eur J Med Chem 276:116699. 10.1016/j.ejmech.2024.116699 39089000 10.1016/j.ejmech.2024.116699

[CR50] Ozgencli I, Budak H, Ciftci M, Anar M (2019) Lichen acids may be used as a potential drug for cancer therapy; by inhibiting mitochondrial thioredoxin reductase purified from rat lung. Anticancer Agents Med Chem 18:1599–1605. 10.2174/1871520618666180525095520 10.2174/1871520618666180525095520 29793415

[CR51] Qian C-N, Mei Y, Zhang J (2017) Cancer metastasis: issues and challenges. Chin J Cancer 36:38. 10.1186/s40880-017-0206-7 28372569 10.1186/s40880-017-0206-7 PMC5379757

[CR52] Rajavel T, Banu Priya G, Suryanarayanan V et al (2019) Daucosterol disturbs redox homeostasis and elicits oxidative-stress mediated apoptosis in A549 cells via targeting thioredoxin reductase by a p53 dependent mechanism. Eur J Pharmacol 855:112–123. 10.1016/j.ejphar.2019.04.051 31059712 10.1016/j.ejphar.2019.04.051

[CR53] Roser LA, Erkoc P, Ingelfinger R et al (2022) Lecanoric acid mediates anti-proliferative effects by an M phase arrest in colon cancer cells. Biomed Pharmacother 148:112734. 10.1016/j.biopha.2022.112734 35190352 10.1016/j.biopha.2022.112734

[CR54] Sahin E, Dabagoglu Psav S, Avan I et al (2019) Vulpinic acid, a lichen metabolite, emerges as a potential drug candidate in the therapy of oxidative stress–related diseases, such as atherosclerosis. Hum Exp Toxicol 38:675–684. 10.1177/0960327119833745 30868920 10.1177/0960327119833745

[CR55] Sharifi-Rad M, Anil Kumar N V., Zucca P, et al (2020) Lifestyle, oxidative stress, and antioxidants: back and forth in the pathophysiology of chronic diseases. Front Physiol 11. 10.3389/fphys.2020.00694 10.3389/fphys.2020.00694 PMC7347016 32714204

[CR56] Solárová Z, Liskova A, Samec M et al (2020) Anticancer potential of lichens’ secondary metabolites. Biomolecules 10:87. 10.3390/biom10010087 31948092 10.3390/biom10010087 PMC7022966

[CR57] Sönmez Aydın F, Hukkamlı B, Budak H (2021) Coaction of hepatic thioredoxin and glutathione systems in iron overload‐induced oxidative stress. J Biochem Mol Toxicol 35. 10.1002/jbt.22704 10.1002/jbt.22704 33393188

[CR58] Studzińska-Sroka E, Majchrzak-Celińska A, Zalewski P et al (2021) Lichen-derived compounds and extracts as biologically active substances with anticancer and neuroprotective properties. Pharmaceuticals 14:1293. 10.3390/ph14121293 34959693 10.3390/ph14121293 PMC8704315

[CR59] Sulukoğlu EK, Günaydın Ş, Kalın ŞN et al (2024) Diffractaic acid exerts anti-cancer effects on hepatocellular carcinoma HepG2 cells by inducing apoptosis and suppressing migration through targeting thioredoxin reductase 1. Naunyn Schmiedebergs Arch Pharmacol 397:5745–5755. 10.1007/s00210-024-02980-5 38308689 10.1007/s00210-024-02980-5 PMC11329542

[CR60] Testa U, Castelli G, Pelosi E (2018) Lung cancers: molecular characterization, clonal heterogeneity and evolution, and cancer stem cells. Cancers (Basel) 10:248. 10.3390/cancers10080248 30060526 10.3390/cancers10080248 PMC6116004

[CR61] Travis WD, Brambilla E, Nicholson AG et al (2015) The 2015 World Health Organization classification of lung tumors. J Thorac Oncol 10:1243–1260. 10.1097/JTO.0000000000000630 26291008 10.1097/JTO.0000000000000630

[CR62] Venkatesan G, Ping CY, Chen H et al (2024) Design, synthesis, molecular docking, and evaluation of sulfonyl quinazoline analogues as promising liver cancer drugs. Bioorg Chem 153:107777. 10.1016/j.bioorg.2024.107777 39244968 10.1016/j.bioorg.2024.107777

[CR63] Wang S, Yan Y, Cheng Z et al (2018) Sotetsuflavone suppresses invasion and metastasis in non-small-cell lung cancer A549 cells by reversing EMT via the TNF-α/NF-κB and PI3K/AKT signaling pathway. Cell Death Discov 4:26. 10.1038/s41420-018-0026-9 29531823 10.1038/s41420-018-0026-9 PMC5841291

[CR64] Wei T, Xiaojun X, Peilong C (2020) Magnoflorine improves sensitivity to doxorubicin (DOX) of breast cancer cells via inducing apoptosis and autophagy through AKT/mTOR and p38 signaling pathways. Biomed Pharmacother 121:109139. 10.1016/j.biopha.2019.109139 31707337 10.1016/j.biopha.2019.109139

[CR65] White P, Oliveira R, Oliveira A et al (2014) Antioxidant activity and mechanisms of action of natural compounds isolated from lichens: a systematic review. Molecules 19:14496–14527. 10.3390/molecules190914496 25221871 10.3390/molecules190914496 PMC6271897

[CR66] Wood SL, Pernemalm M, Crosbie PA, Whetton AD (2014) The role of the tumor-microenvironment in lung cancer-metastasis and its relationship to potential therapeutic targets. Cancer Treat Rev 40:558–566. 10.1016/j.ctrv.2013.10.001 24176790 10.1016/j.ctrv.2013.10.001

[CR67] Wu Y, Gan D, Leng X et al (2022) Anti-ageing and anti-lung carcinoma effects of vulpinic acid and usnic acid compounds and biological investigations with molecular modeling study. J Oleo Sci 71:ess21276. 10.5650/jos.ess21276 10.5650/jos.ess21276 35110467

[CR68] Yangın S, Cansaran-Duman D, Eskiler GG, Aras S (2022) The molecular mechanisms of vulpinic acid induced programmed cell death in melanoma. Mol Biol Rep 49:8273–8280. 10.1007/s11033-022-07619-3 35960408 10.1007/s11033-022-07619-3

[CR69] Yu J, Zhong B, Xiao Q et al (2020) Induction of programmed necrosis: a novel anti-cancer strategy for natural compounds. Pharmacol Ther 214:107593. 10.1016/j.pharmthera.2020.107593 32492512 10.1016/j.pharmthera.2020.107593

[CR70] Zhang J, Yao J, Peng S et al (2017) Securinine disturbs redox homeostasis and elicits oxidative stress-mediated apoptosis via targeting thioredoxin reductase. Biochim Biophys Acta - Mol Basis Dis 1863:129–138. 10.1016/j.bbadis.2016.10.019 27777067 10.1016/j.bbadis.2016.10.019

